# Monosodium urate crystals induce extracellular DNA traps in neutrophils, eosinophils, and basophils but not in mononuclear cells

**DOI:** 10.3389/fimmu.2012.00277

**Published:** 2012-09-03

**Authors:** Christine Schorn, Christina Janko, Melanie Latzko, Ricardo Chaurio, Georg Schett, Martin Herrmann

**Affiliations:** Department of Internal Medicine 3, Institute for Clinical Immunology, Friedrich-Alexander University Erlangen-NurembergErlangen, Germany

**Keywords:** neutrophil extracellular traps, NETs, MSU, granulocytes, bacteria, PMA, inflammation

## Abstract

Neutrophil extracellular traps (NETs) are fibers of extracellular DNA released from neutrophils due to overwhelming phagocytic stimuli. The function of NETs is to trap and kill microbes to avoid spreading of potential pathogens. NETs are formed after encounter with various gram-positive and -negative bacteria but also in response to mediators causing sterile inflammation like interleukin-8 (IL-8), tumor necrosis factor (TNF), and phorbol myristate acetate (PMA). Here we show the formation of NETs (NETting) in response to monosodium urate (MSU) crystals as further model for sterile inflammation. We identified monocytes, neutrophils, and eosinophils as MSU phagocytosing cells. Basophils did not take up the crystals, instead they upregulated their activation marker CD203c after contact with MSU. Nevertheless, MSU crystals induced extracellular trap formation also in basophils, like in eosinophils and neutrophils, which phagocytose the crystals. In contrast, monocytes do not form NETs despite uptake of the MSU crystals. In contrast to the canonical stimuli like bacteria and PMA, MSU-induced NETosis was not abrogated by plasma. Our data show that MSU crystals induce extracellular DNA trap formation in all three granulocytes lineages (NETs, EETs, and BETs) but not in monocytes, and DNA externalization does not necessitate the uptake of the crystals.

## Introduction

Granulocytes play a crucial part in the innate immunity and can be subdivided into, neutrophils, eosinophils, and basophils with different features and functions for the immune response. Eosinophils develop from myeloid precursor cells in bone marrow stimulated by the cytokines IL-3, IL-5, and GM-CSF (Clark et al., 2007; von Kockritz-Blickwede et al., 2008; Brill et al., 2012). After maturation, eosinophils circulate in the blood and migrate to inflammatory sites in the tissues, or to helminth infections in response to chemokines like RANTES, and certain leukotrienes like LTB4. Following activation by type 2 cytokines released from Th2 cells, eosinophils produce ROS (Cline et al., 1968), growth factors (Rothenberg and Hogan, 2006) and cytokines (Crivellato et al., 2010), degranulate and release lipid mediators (Chirumbolo, 2012) and cytotoxic granule proteins (Popa-Nita and Naccache). Granule proteins from eosinophils include major basic protein, eosinophil cationic protein, and eosinophil peroxidase which can cause cell death in the target cell by induction of toxic pores and oxidative stress, but also may induce tissue damage and dysfunction (Crivellato et al., 2010). Major basic protein induces degranulation in mast cells and basophils (Zheutlin et al., 1984), which are together with eosinophils mediators of allergic responses and asthma pathogenesis.

Basophils are circulating granulocytes that orchestrate hypersensitivity (atopic) and anaphylactic reactions (Simons, 2010) and share a common lineage with tissue-dwelling mast cells. Recently basophils got into the focus due to their strategic role linking innate and acquired immunity. Besides promoting chronic inflammation in allergy (Mukai et al., 2009), basophils regulate Th2 cell function (Sokol and Medzhitov, 2010) immune cell memory and even serve as antigen-presenting cells (Maddur et al., 2010). In case of an infection, mature basophils degranulate and release histamine, proteoglycans (both prestored), the cytokines IL-3, IL-4, IL-6, IL-9, IL-13, IL-25, and GM-CSF, proteolytic enzymes, lipid mediators, and the chemokines MCP-1, MIP-1α, MIP-1β, and RANTES. However, basophils do not release the immunregulatory cytokines IFN-γ, IL-17, or IL-5 (Schroeder et al., 2009; Yamaguchi et al., 2009).

During the acute phase of infection neutrophils get attracted by chemotactic molecules, leave the blood vessels and migrate toward the site of infection (Massena et al., 2010). These phagocytic cells can incorporate microorganisms and kill them via antimicrobial proteins, proteolytic enzymes, and reactive oxygen species (ROS) (Clifford and Repine, 1982; Borregaard and Cowland, 1997). In addition, an alternative extracellular killing mechanism has been described when the phagocytic capacity is exhausted (Brinkmann et al., 2004; Urban et al., 2006). Activated neutrophils form extracellular fibers, called neutrophil extracellular traps (NETs), that are composed of loosened chromatin and granule proteins such as neutrophil elastase, cathepsin G and lactotransferrin (Urban et al., 2009). NETs immobilize the pathogens and, thereby, prevent microbial spreading (Brinkmann et al., 2004; Urban et al., 2006). NETs containing antimicrobial molecules efficiently kill microorganisms and contribute to an anti-microbial environment (Urban et al., 2006). The molecular binding mechanism is still unknown, but electrostatic interactions between the anionic surface of the microorganisms and cationic components of the NETs are discussed (Brinkmann and Zychlinsky, 2007). Formation of NETs (NETting) causes neutrophils' death, but NETosis is an active cell death mechanism distinct from apoptosis and necrosis and depends on the production of ROS by NADPH-oxidase (Fuchs et al., 2007). Blocking the respiratory burst by diphenylene iodonium (DPI) inhibits NETs formation. Patients suffering from chronic granulomatous disease (CGD) carry mutations in the phagocyte NADPH oxidase and are unable to generate ROS and to form NETs (Fuchs et al., 2007). After ROS production in activated neutrophils the nuclear membranes disintegrate generating vesicles and nuclear material as well as granular components are mixed (Brinkmann and Zychlinsky, 2007). Finally cells break off and release the NETs. NETting in granulocytes can be induced in response to various gram-positive and -negative bacteria as well as fungi and parasites but also by sterile mediators as interleukin-8 (IL-8), tumor necrosis factor (TNF) and phorbol myristate acetate (PMA) (von Kockritz-Blickwede and Nizet, 2009).

The deposition of monosodium urate (MSU) crystals in tissues and joints causes gouty arthritis. When uric acid, the final product of the human purine metabolism, exceeds the limit of solubility (70 μg/ml), it crystallizes as sodium containing MSU (So, 2007; Schorn et al., 2011). The uptake of MSU crystals is accompanied by the release of pro-inflammatory cytokines and chemokines from granulocytes and monocytes (Schorn et al., 2010).

In our study we employed MSU as further model for sterile inflammation. We analyzed the effect of MSU crystals on in all three granulocytes lineages (neutrophils, eosinophils, and basophils) and mononuclear cells (monocytes, B cells, and T cells). We observed that monocytes, neutrophils, and eosinophils take up MSU crystals. Although basophils did not phagocytose MSU crystals, they upregulated their lineage marker CD203c after contact with MSU. Furthermore, we identified neutrophils, eosinophils, and basophils as cells capable to externalize nuclear DNA after incubation with MSU crystals. In contrast to NETting induced by PMA and bacteria, the formation of MSU-dependent NETs was not inhibited by plasma.

## Materials and methods

### Isolation of PMN, PBMC, eosinophils, and basophils from human whole blood

Venous blood was taken from human blood donors in full agreement with institutional guidelines. Heparinized whole blood (20 U/ml) was centrifuged at 3400 g for 10 min (Rotina 46, Hettich) for the generation of autologous plasma. Peripheral blood mononuclear cells (PBMC) and polymorphonuclear neutrophils (PMN) were purified by density gradient centrifugation using Lymphoflot (Bio-Rad). The PBMC fraction was purified from platelets by centrifugation through a cushion of fetal bovine serum (Invitrogen GmbH). Erythrocytes were eliminated by hypotonic lysis. Basophils were negatively isolated from the PBMC fraction via indirect magnetic cell sorting employing a Basophil Isolation Kit (Miltenyi Biotech). The purity of the isolated basophils was verified by CD123/CD303 staining. Eosinophils were achieved from the PMN fraction employing an Eosinophil Isolation Kit (Miltenyi Biotech) according to the standard protocol. The purity of the isolated eosinophils was assured by quantifying CD16^−^CD49d^+^ cells. Viable cells were counted in a Neubauer counting chamber and cell density was adjusted to 2 × 10^6^ cells/ml in 100% autologous plasma.

### Fluorescence microscopy

Whole blood cells or isolated cells were incubated for 2–5 h with 1 mg/ml or 200 μg/ml MSU crystals, respectively. Cytospins were prepared after lysing erythrocytes and non-ingested crystals by the TQprep Workstation (Beckman Coulter). The nuclear and extranuclear DNA of the samples was stained with 1 μg/ml 4′-6-Diamidino-2-phenylindole (DAPI; Invitrogen GmbH) for 30 min. After washing, the samples were analyzed by fluorescence microscopy using standard filter sets. For a better demonstration NETs and cells were artificially colored by morphology of their nucleus/DNA distribution. NETs were colored in green, polymorphonuclear cells in red, mononuclear cells in blue and MSU crystals in yellow or white.

For the detection of nuclear histone H3 in NETs, a monoclonal anti-human histone H3 antibody (1.5 μg/ml) produced in mice (Abcam) was incubated with cytospins for 30 min at room temperature, washed twice with PBS and incubated with goat anti-mouse IgG-FITC (Southern Biotech), washed with PBS and analyzed by fluorescence microscopy.

### Analyzes of MSU phagocytosing cells

Whole blood cells were incubated with 1 mg/ml MSU crystals for 1 h at 37°C. The phagocytosis of MSU crystals was determined by analyzing the SSc change in flow cytometry. We identified the following cell types by morphological properties (FSc, SSc) and by cell specific surface markers: CD14^+^ monocytes, CD16^+^ neutrophils, CD16^−^CD49d^+^ eosinophils, CD123^+^CD203^+^ basophils, BDCA-2^+^CD11c^−^ plasmacytoid dendritic cells (pDC), CD3^+^CD56^−^ T cells, CD3^−^CD56^−^ B cells, CD3^−^CD56^+^ natural killer (NK) cells, and CD3^+^Cd56^+^ natural killer T cells (NKT cells).

### Plasma concentration dependency of NETting PMN

PMN were isolated from human venous blood as described previously. We investigated 5 × 10^6^ cells/ml in 0% plasma (100% R0), 20% plasma (+80% R0), and 100% plasma, whereas R0 was composed of RPMI 1640 medium (Gibco) with 1% penicillin/streptomycin (Gibco), 1% hepes (Merck), and 1% glutamine (Gibco). NETs formation was stimulated by 100 nM PMA, 2 × 10^7^ bacteria (heat inactivated streptococcus pneumoniae), 200 μg/ml MSU crystals or R0 as control for 4 h at 37°C. After preparation of cytospins, the DNA was stained with 1 μg/ml DAPI and the NETs formation was analyzed by fluorescence microscopy.

### Quantification of cytokines/chemokines

The concentration of certain chemokines/cytokines was analyzed in culture supernatants of 2 × 10^6^ isolated eosinophils, basophils and neutrophils per milliliter in autologous plasma after 18 h incubation with 200 μg/ml MSU crystals or PBS as control employing multiplex bead technology (eBioscience). Quantification of cytokines/chemokines was gained by cytofluorometry.

### Cytospins

2 × 10^5^ cells were centrifuged onto glass slides (ThermoFisher) at 850 g for 10 min (Rotina 46, Hettich) using cytospin cuvettes. The supernatant was removed and the slides were centrifuged for additional 5 min at 2000 g. The DNA of PFA-fixed cells was stained with 1 μg/ml DAPI and analyzed by fluorescence microscopy using standard filter sets.

### Preparation of MSU crystals

A solution of 10 mM uric acid and 154 mM NaCl (both from Merck KGaA) was adjusted to pH 7.2 and agitated for 3 days for the production of MSU crystals. For sterilization the needle-shaped crystals were washed with ethanol, dried under sterile conditions and heated at 180°C for 2 h. The crystals were stored in 40 mg/ml in sterile PBS. In whole blood assays we used 1 mg/ml and in analysis with isolated cells we used 200 μg/ml MSU crystals. The limit of solubility of MSU crystals is 70 μg/ml.

### Lysis of erythrocytes, solubilization of crystals, and flow cytometry

Erythrocytes and non-ingested MSU crystals were automatically lysed using a TQprep Workstation (Beckman Coulter) before measurement with a Gallios™ cytofluorometer (Beckman Coulter). The cytofluorometric data were analyzed with the Kaluza software (Beckman Coulter). Electronic compensation was used to eliminate bleed-through fluorescence.

### Statistical analysis

We performed statistical analyzes with SPSS PASW statistics 18. The results are represented as mean ± SEM of at least three and up to five independent experiments. Student's *t*-test or an analysis of variance for repeated measurements was used. The alpha level of all the tests or the P value was set at 0.05.

## Results

### MSU crystals induce formation of extracellular traps by granulocytes

**Figure 1:**
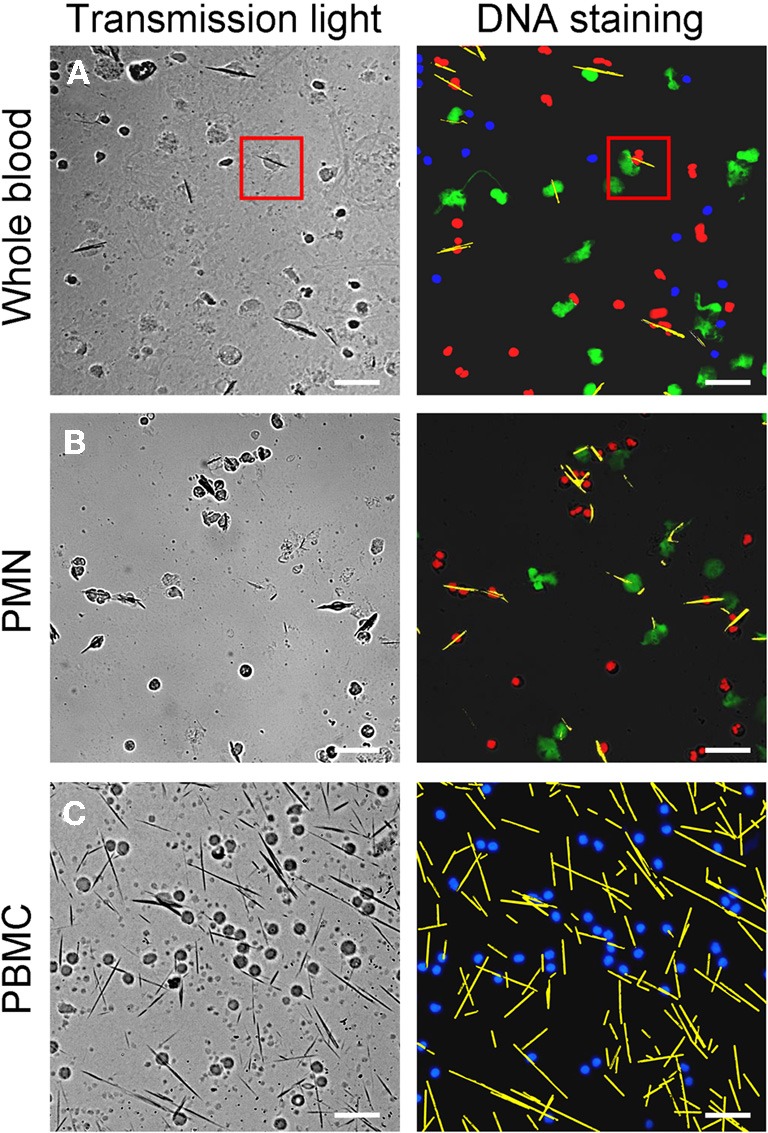
**MSU crystals induce NET formation in PMN but not in PBMC.** Whole blood cells **(A),** isolated PMN **(B)** and isolated PBMC **(C)** were incubated with MSU crystals. Cytospins were prepared and stained for nuclear and extranuclear DNA. The transmission microscopy (left) shows phagocytes which have ingested MSU crystals. The DNA staining (right) shows NETting granulocytes in whole blood cells (**A**, red square) and isolated PMN **(B)**. No MSU-induced extracellular DNA was observed in isolated PBMC **(C)**. In **(A)** and **(B)** erythrocytes and free MSU crystals were dissolved by acid treatment. **(A–C)** NETs were artificial colored in green, PMN in red, mononuclear cells in blue and MSU crystals in yellow. All experiments were performed at least 3 times. Scale bars, 100 μm.

**Figure 2:**
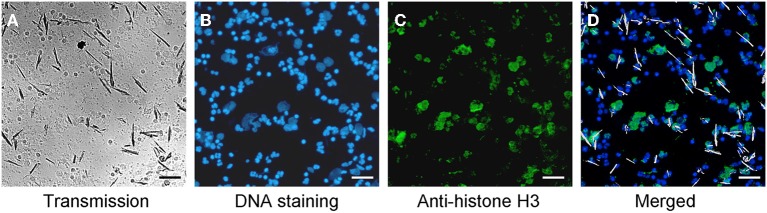
**MSU induced NETs contain histon H3. (A–D)** Whole blood cells were incubated with MSU crystals. Cytospins were prepared and stained for nuclear and extranuclear DNA **(B)** and histon H3 **(C)**. The merged picture **(D)** shows the co-localization of DNA and histon H3. Erythrocytes and free MSU crystals were dissolved by acid treatment. The MSU crystals were artificial colored in white. All experiments were performed at least 3 times. Scale bars, 100 μm.

After incubation of whole blood with MSU, we detected extranuclear DNA in the cultures (Figure 1A). Employing transmission light microscopy of cytospins we observed that crystal-speared phagocytes (Figure 1A, left) were surrounded by externalized DNA (Figure 1A, right). After identification of NETs formation by blood cells after contact with MSU crystals, we analyzed the NETting capacity of isolated PMN and PBMC. Figure 1B shows that neutrophils ejected NETs (right) after phagocytosis of MSU crystals (left). The residual MSU crystals are trapped and immobilized by the NETs. In contrast to PMN, isolated PBMC did not respond with the formation of extracellular traps, suggesting that the latter skill is confined to granulocytes (Figure 1C). Furthermore, the existence of NETs induced by MSU crystals was confirmed by histon H3 staining (Figures 2A–D).

### Monocytes, neutrophils, and eosinophils take up MSU crystals

**Figure 3:**
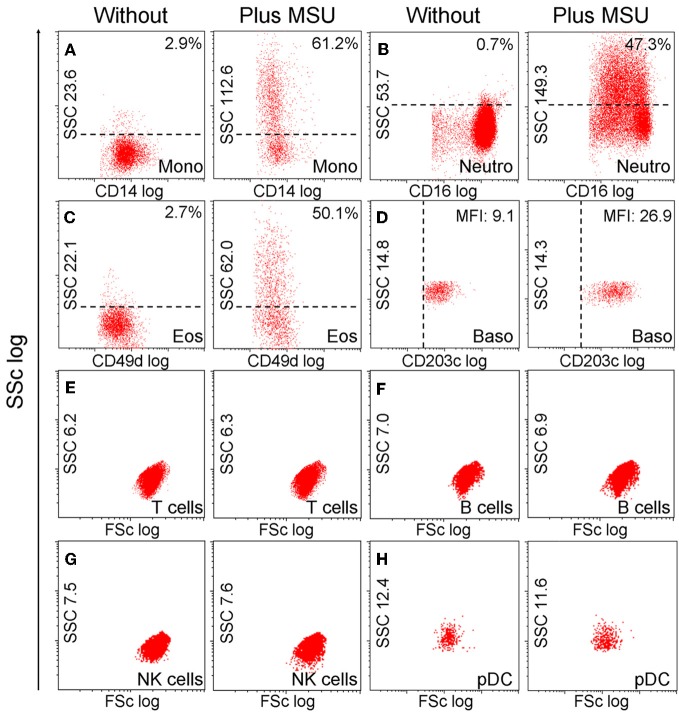
**Monocytes, neutrophils and eosinophils ingest MSU crystals. (A–H)** Whole blood cells were incubated with MSU crystals. After lysis of the erythrocytes the morphology (forward scatter, side scatter) of the leukocytes was characterized by flow cytometry. The individual leukocyte populations were defined by cell lineage-specific surface markers. The uptake of crystals was reflected by dramatic increases of the cells' SSc values after phagocytosis. Monocytes **(A)** neutrophils **(B)** and eosinophils **(C)** ingested MSU crystals. Basophils **(D)** did not take up the crystals, but upregulated their surface activation marker CD203c. T cells **(E)** B cells **(F)** NK cells **(G)** and pDCs **(H)** did not increase the side scatter in response to MSU crystals. SSc, side scatter; FSc, forward scatter; Mono, monocytes; neutro, neutrophils; eos, eosinophils; baso, basophils; NK, natural killer cells; pDC, plasmacytoid dendritic cells. All experiments were performed at least three times.

Next we analyzed the cell types able to ingest MSU crystals. For this purpose, we incubated whole blood with MSU crystals and subsequently stained the cells with marker antibodies to identify the cell types involved in MSU clearance. The cell types were identified in flow cytometer according to their morphology (forward and side scatter reflect information about cell size and granularity, respectively) and surface markers (Figure 3). The uptake of MSU crystals by monocytes (61.2%), neutrophils (47.3%), and eosinophils (50.1%) was reflected by a drastically increased side scatter (SSc) of cells with the surface markers CD14, CD16, and CD49d, respectively. In contrast, basophils (CD203c^+^ cells) did not increase their side scatter but upregulated their lineage marker CD203c after incubation with MSU crystals. B cells, T cells, NK cells, pDC, (Figure 3) and NKT cells (not shown) did not respond to MSU crystals with an increase in the SSc.

### Neutrophils, eosinophils, and basophils form extracellular traps after incubation with MSU crystals

**Figure 4:**
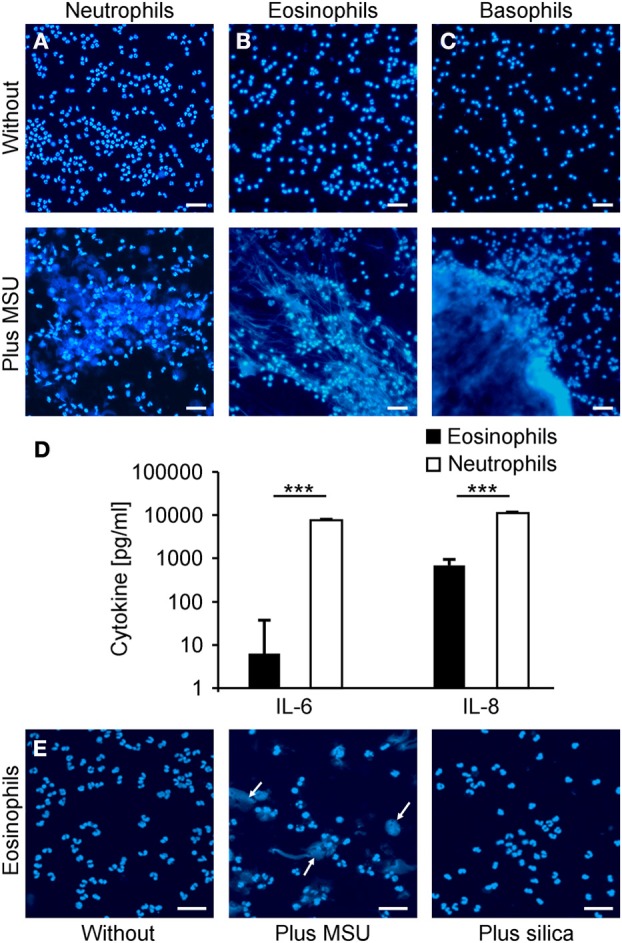
**Neutrophils, eosinophils and basophils form extracellular traps after incubation with MSU. (A–C)** Isolated neutrophils **(A)**, eosinophils **(B)** or basophils **(C)** in plasma were incubated without (upper) or with MSU crystals (lower). Cytopsins were prepared and stained for extranuclear DNA. The DNA staining showed extracellular trap formation and clumping in neutrophils, eosinophils and basophils. **(D)** Cytokine/chemokine induction of eosinophils and neutrophils were analyzed after MSU incubation. Eosinophils produce traces of IL-6 and intermediate amounts of IL-8 after MSU incubation. Neutrophils release high amounts of IL-6 and IL-8 after incubation with MSU crystals. Values of mock-treated cells served as baseline. **(E)** Isolated eosinophils were incubated without (left), with MSU crystals (middle), or with silica crystals (right). The DNA staining shows extracellular trap formation (arrows) only in samples with MSU crystals. All experiments were performed at least three times. Scale bars, 100 μm; ^***^P < 0.001.

Neutrophils have been described to externalize nuclear DNA that forms NETs in response to various stimuli. Since the NET induction by MSU was faster and more efficient than canonical activators of NETting we analyzed the potential to externalize nuclear DNA of neutrophils, eosinophils, basophils, and monocytes in response to MSU. To this end we employed isolated neutrophil, eosinophil, and basophil granulocytes as well as PBMC to study the clearance of MSU. Figure 4 shows that neutrophils (Figure 4A), eosinophils (Figure 4B) and basophils (Figure 4C) release extracellular DNA after culture in the presence of MSU crystals. Monocytes did not form extracellular traps despite the uptake of crystals (not shown). Furthermore, MSU crystals induced the release of IL-8 in the supernatants of eosinophils. In contrast, neutrophils released huge amounts of IL-6 and IL-8 during NET formation (Figure 4D). Since basophils are well known to degranulate spontaneously during prolonged culture the baseline cytokine levels were very high and no cytokine response to MSU was to be observed (not shown). The formation of extracellular traps by eosinophils was specific for MSU crystals; silica crystals did not induce EET structures (Figure 4E).

### NETting of granulocytes induced by MSU is not sensitive to plasma

**Figure 5:**
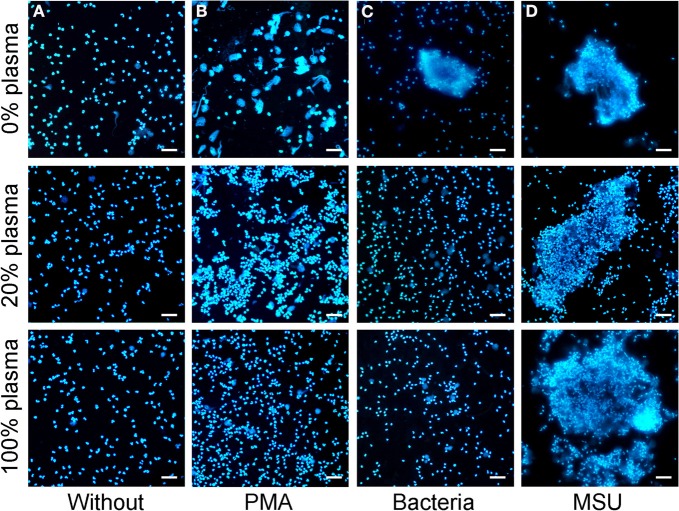
**MSU induced NETting of neutrophils is not inhibited by plasma. (A–D)** Isolated neutrophils were incubated without stimuli **(A)**, with PMA **(B)**, with bacteria **(C)**, or with MSU crystals **(D)** in the presence of different concentrations of plasma (0% upper; 20% middle, 100% lower). High plasma concentrations inhibit NETting induced by PMA **(B)** and by bacteria **(C)**. MSU induced NETting **(D)** is not inhibited and rather increased by high concentrations of plasma. All experiments were performed at least three times. Scale bars, 100 μm.

The induction of PMA induced NETs is reportedly inhibited even by low concentrations of plasma and is fully abrogated by high plasma concentrations (> 20%). Therefore, we analyzed the NETs formation by PMN in the presence of plasma (Figure 5). We incubated isolated PMN with PMA or bacteria, both canonical stimuli for NETs formation, or with MSU crystals in medium containing 0%, 20%, or 100% plasma. PMA, bacteria and MSU crystals induced NET formation in the absence of plasma as revealed by DNA staining. NETs formation induced by bacteria and PMA was drastically reduced in the presence of 20% or 100% plasma. In contrast, MSU crystals induced large NETs by PMN even in the presence of pure plasma. The size and density of the NET aggregates was even higher in the presence of increasing amounts of plasma (Figure 5).

## Discussion

Neutrophils have been identified by several groups to produce extracellular DNA traps and consequently these structures have been named “neutrophil extracellular traps.” Recent data indicate that this phenomenon is not restricted to neutrophils but is also executed by other cells (Guimaraes-Costa et al., 2012). Beside neutrophils, mast cells and eosinophils have already been described to produce extracellular traps (von Kockritz-Blickwede et al., 2008). In this manuscript we describe a systematic analysis of the ability of blood leukocytes to release extracellular DNA in response to MSU crystals, a strong stimulus of sterile inflammation. In accordance with previous data investigating canonical inducers of NET formation, like PMA, bacteria, and LPS (Fuchs et al., 2007) we observed that PBMC do not release NETs after culture with MSU crystals.

We found that neutrophils, eosinophils, and basophils react differentially in response to MSU crystals. Neutrophils and eosinophils both ingest MSU and release nuclear DNA that forms extracellular fibers. However, in whole blood not all cells may get in direct contact to the crystals within the time frame of the assay (1 h), and do not increase their side scatter. After longer incubations, the NETosis sequesters the crystals in dense clots. Interestingly, the basophils did not ingest the MSU, but got activated as shown by expression of the activation marker CD203c. Two hours after contact to the MSU crystals, all three granulocytes subtypes formed clusters composed of externalized DNA densely associated with the crystals. In the blood these aggregates were specific for granulocytes and were never seen with other cell types such as monocytes or lymphocytes. Interestingly, as noticed in previous works the formation of extracellular traps was independent of the ingestion of MSU crystals.

Neutrophils are the major leucocyte population of blood. Within minutes following trauma, neutrophils migrate toward the site of inflammation, where they are involved in the frontline immune defense, in phagocytosis of pathogens and in the formation of pus (Weiss, 1989). Neutrophil phagocytosis is strongly enhanced by opsonization of their prey (Burnett et al., 1993). After internalization they may kill the microbes by the formation of ROS and hydrolytic enzymes. Alternatively, neutrophils may degranulate and release an assortment of proteins from specific, azurophilic and tertiary granules (Kumar and Sharma, 2010). In 2004, Brinkmann and colleagues described a surprising observation: certain modes of neutrophil activation cause the release of net-like DNA structures into the extracellular space (Brinkmann et al., 2004). These represent a third mechanism for trapping and killing microbes independent of phagocytosis. This mechanism mainly works extravascularly since NET formation and agglutination of bacteria within the vessels may carry the risk for thrombotic events (Brill et al., 2012) Therefore, it is not surprising that plasma is a strong inhibitor for NETting (Fuchs et al., 2007). Under extreme conditions like sepsis, rapid intravascular NET formation has been observed in the liver sinusoids and the pulmonary capillaries, where it occurs at the expense of endothelial injury (Clark et al., 2007). Similar to this life-threatening condition, the stimulus exerted by MSU crystals also overcomes the inhibitory activity of plasma. Whether this contributes to the pathogenesis of gout is currently under investigation.

Eosinophils ingest inert polystyrene particles and bacteria although less efficiently than neutrophils. The concomitants of phagocytosis, including transcriptional activity, secretion of enzymes and degranulation are similar in neutrophils and eosinophils (Cline et al., 1968). Eosinophils are involved in the fight against helminth infections and are also elevated in the presence of certain parasites. A role for eosinophils in other biological processes has been reported for e.g., allograft rejection and neoplasia (Rothenberg and Hogan, 2006). Therefore, the formation of extracellular traps may contribute to the function of the eosinophils by immobilizing their targets (Guimaraes-Costa et al., 2012). Similar to neutrophils, eosinophils release cytokines during the formation of the extracellular DNA traps. Here, a bias by contaminating neutrophils in the eosinophil preparation is unlikely since the cytokine profiles of eosinophils and neutrophils differed considerably.

Like neutrophils and eosinopils, basophils are motile cells that migrate into inflamed tissues. Similar to mast cells basophils are prone to orchestrate tissue healing and inflammation-related angiogenesis; the latter through the action of vascular endothelial growth factors and their receptors (Crivellato et al., 2010). The failure to identify basophil-derived cytokines and chemokines induced by MSU crystals is due to the well-established tendency of these cells for spontaneous degranulation (Chirumbolo, 2012), which resulted in high baseline levels of these mediators blurring MSU specific effects.

The uptake of MSU crystals by phagocytes has been shown to be dependent on heat-labile serum-factors and divalent cations (Schorn et al., 2010). In eosinophils, a catapult-like release of mitochondrial DNA after stimulation with lipopolysaccharide, complement or eotaxin has been described, which, reportedly did not end up in cell death (Nizet and Rothenberg, 2008). This is in striking contrast to the formation of extracellular DNA aggregated by eosinophils after coculture with MSU crystals. Here the externalization of nuclear DNA clearly indicated cell death. We also found an upregulation in the release of proinflammatory cytokines by eosinophils after challenge with MSU. These crystals, being responsible for gout, robustly externalized DNA in all subtypes of granulocytes e.g., NETs, EETs, and BETs for neutrophils, eosinophils and basophils, respectively.

Compared with stimuli like PMA or bacteria, which elicit NETs only in conditions of low plasma, MSU provoked NETs also in the presence of full plasma. We argue that the stimulus exerted by MSU is more robust than those from the canonical inducers bacteria and PMA. However, the exact definition of the differences needs further examination. The wide extension of the MSU-induced NETs is most likely due to the fact that the crystals act as scaffold stabilizing the extracellular DNA. Neutrophils are known to be critical for gouty inflammation (Popa-Nita and Naccache, 2010), and NETs have been identified in cells from the synovial fluid (Mitroulis et al., 2011). Pro-inflammatory cytokines carrying danger signals, and protolytical enzymes attached to the NETs may promote tissue injury and inflammation in gouty arthritis. A role of NETs has also been described for the pathogenesis of SLE and asthma, suggesting an importance of these extracellular DNA structures in the amplification of inflammatory responses (Hakkim et al., 2010). In the case of SLE, the well-established deficiency for the clearance of dying cells and nuclear remnants also includes the dismantling of NETs (Janko et al., 2008; Muñoz et al., 2010).

### Conflict of interest statement

The authors declare that the research was conducted in the absence of any commercial or financial relationships that could be construed as a potential conflict of interest.

## Abbreviations

BET: basophil extracellular trap
DAPI: 4′-6-Diamidino-2-phenylindole
EET: eosinophil extracellular trap
FSc: forward scatter
IL: interleukin
LPS: lipopolysaccharide
MSU: monosodium urate
NET: neutrophil extracellular trap
PBMC: peripheral blood mononuclear cells
PFA: paraformaldehyde
PMA: phorbol myristate acetate
PMN: polymorphonuclear cells
ROS: reactive oxygen species
SSc: side scatter.
